# Evaluation of serum vitamin D levels and their impact on functional outcomes among patients undergoing total knee arthroplasty

**DOI:** 10.6026/973206300213819

**Published:** 2025-10-31

**Authors:** Mohammed Kinaan Khalid, Vishnu Jayaprakasan, Rohan Ghosh

**Affiliations:** 1 Department of Emergency Medicine, Syrus MM Hospital, Kannur, Kerala, India; 2 Department of Orthopaedics, Kannur Medical College Anjarakkandy, Kannur, Kerala, India; 3 Department of Emergency Medicine, Anandaloke Multispeciality Hospital, Sevoke Road, Siliguri, West Bengal, India

**Keywords:** Vitamin D, total knee arthroplasty, functional outcomes, KSS, WOMAC, deficiency

## Abstract

Vitamin D deficiency is common among patients undergoing total knee arthroplasty (TKA) and may adversely affect postoperative recovery. This prospective study of 120 patients evaluated the association between preoperative serum vitamin D levels and functional outcomes using KSS and WOMAC scores. Patients with sufficient vitamin D (≥30 ng/mL) showed significantly better KSS (85.2 ± 6.3) and lower WOMAC (18.3 ± 5.2) scores at 6 months compared to deficient individuals (p < 0.001). Vitamin D deficiency remained an independent predictor of poorer recovery after adjustment for confounders. Preoperative correction of vitamin D deficiency may improve functional outcomes following TKA.

## Background:

Total knee arthroplasty (TKA) is a highly effective intervention for end-stage knee osteoarthritis, with over 700,000 procedures performed annually in the United States alone [1]. Despite high implant survival rates, functional outcomes vary significantly, with 15-30% of patients reporting persistent pain or limited mobility [2]. Recent evidence suggests that nutritional status, particularly vitamin D levels, may influence postoperative recovery [3]. Vitamin D, a fat-soluble hormone critical for bone mineralization, muscle function and immune modulation, is deficient in 40-80% of orthopedic patients [4]. The significance of vitamin D in TKA outcomes stems from its role in musculoskeletal health. Deficiency is linked to impaired muscle strength, prolonged inflammation and delayed fracture healing [5]. Several studies have reported associations between low vitamin D and increased postoperative pain, slower rehabilitation and higher revision rates in joint arthroplasty [6, 7]. However, conflicting data exist, with some studies failing to demonstrate significant correlations [8]. This discrepancy may arise from heterogeneity in outcome measures, follow-up durations and inadequate control for confounders such as age, body mass index (BMI) and comorbidities [9]. Recent investigations have highlighted the potential benefits of vitamin D repletion in surgical populations. A 2021 meta-analysis concluded that supplementation improved functional scores in hip fracture patients [10], but analogous data in TKA remain limited. Moreover, no studies have comprehensively evaluated vitamin D's impact on validated functional metrics like KSS and WOMAC across multiple postoperative time points while adjusting for key covariates [11]. This gap impedes clinical guidelines for preoperative nutritional optimization. Therefore, it is of interest to prospectively evaluate the association between preoperative serum vitamin D levels and functional outcomes in patients undergoing TKA. We hypothesized that vitamin D deficiency would independently predict poorer functional recovery at 3 and 6 months postoperatively.

## Materials and Methods:

## Study design:

A prospective cohort study conducted at a tertiary-care orthopedic center between January and December 2023.

## Sample size:

Based on a power analysis (α=0.05, power=80%, effect size=0.5), 120 patients were required to detect clinically significant differences in KSS between vitamin D groups, accounting for a 15% attrition rate.

## Inclusion criteria:

[1] Adults (>18 years) scheduled for primary unilateral TKA for osteoarthritis.

[2] Ability to provide informed consent and complete follow-up assessments.

## Exclusion criteria:

[1] Revision TKA, inflammatory arthritis (e.g., rheumatoid arthritis), or metabolic bone disease.

[2] Renal failure (eGFR <30 mL/min), liver cirrhosis, or malabsorption syndromes.

[3] Current vitamin D supplementation (>1000 IU/day) or bisphosphonate use.

[4] Inability to ambulate preoperatively or attend follow-up visits.

## Tools and procedures:

[1] Vitamin D measurement: Fasting serum samples were collected preoperatively (within 4 weeks of surgery). 25(OH)D levels were quantified via chemiluminescent immunoassay (DiaSorin LIAISON®). Patients were categorized as deficient (<20 ng/mL), insufficient (20-29.9 ng/mL), or sufficient (≥30 ng/mL) per Endocrine Society guidelines [12].

[2] Functional outcomes:

1) Knee Society Score (KSS): Assesses knee (0-100) and function (0-100) domains, with higher scores indicating better function.

2) WOMAC: Evaluates pain (0-20), stiffness (0-8) and physical function (0-68), with total scores ranging from 0-96 (lower = better). Outcomes were measured at baseline (preoperative), 3 months and 6 months postoperatively by blinded physiotherapists.

[3] Covariates: Age, sex, BMI, Charlson Comorbidity Index (CCI) and season of blood draw (winter: November-April; summer: May-October) were recorded.

## Statistical analysis:

Data were analyzed using SPSS v28.0 (IBM Corp.). Continuous variables were expressed as mean ± standard deviation (SD); categorical variables as frequencies (%). Group comparisons used ANOVA for continuous data and chi-square tests for categorical data. Repeated-measures ANOVA assessed changes in functional scores over time. Multiple linear regression models adjusted for confounders to identify independent predictors of 6-month outcomes. Statistical significance was set at p<0.05.

## Results:

Of 120 enrolled patients, 108 (90%) completed 6-month follow-up. Mean age was 65.2 ± 8.5 years and 60% were female. Mean serum 25(OH)D was 22.4 ± 8.1 ng/mL. Deficiency was present in 45% (n=54), insufficiency in 35% (n=42) and sufficiency in 20% (n=24). Groups were comparable in age, sex, BMI and comorbidities (Table 1 - see PDF). All groups improved in KSS and WOMAC over time (p<0.001). However, the sufficient group demonstrated significantly greater improvements than deficient/insufficient groups at 3 and 6 months (Table 2 - see PDF). At 6 months, sufficient patients had higher KSS knee (85.2 ± 6.3 vs. 75.4 ± 7.1; p<0.001) and function scores (83.6 ± 6.9 vs. 72.1 ± 7.5; p<0.001) and lower WOMAC total scores (18.3 ± 5.2 vs. 28.7 ± 6.4; p<0.001) compared to deficient patients. After adjusting for age, sex, BMI and CCI, vitamin D deficiency independently predicted poorer 6-month outcomes. Each 10 ng/mL increase in 25(OH)D was associated with a 3.2-point increase in KSS knee (95% CI: 1.8-4.6; p<0.001) and a 4.5-point decrease in WOMAC total (95% CI: -6.2 to -2.8; p<0.001) (Table 3 - see PDF).

## Discussion:

This study demonstrates that preoperative vitamin D deficiency is prevalent (45%) in TKA candidates and independently predicts poorer functional outcomes at 6 months. Patients with sufficient vitamin D (≥30 ng/mL) achieved significantly higher KSS and lower WOMAC scores than deficient counterparts, even after adjusting for age, sex, BMI and comorbidities. These findings align with emerging evidence linking vitamin D to musculoskeletal recovery in surgical populations [13]. Peng *et al.* [14] conducted a meta-analysis comparing patellofemoral arthroplasty (PFA) vs total knee arthroplasty (TKA) for isolated patellofemoral osteoarthritis and found that while both procedures improved clinical outcomes, PFA yielded better functional recovery and activity levels in the short term. Deering *et al.* [15] performed a systematic review and meta-analysis of tourniquet use in total knee arthroplasty and concluded that tourniquet use was associated with increased postoperative pain without a consistent benefit in range of motion or hospital stay. Paul *et al.* [16] investigated outcomes in patients younger than 55 undergoing primary knee arthroplasty and reported that although implant survivorship remains acceptable, functional outcomes and activity participation are increasingly important in this younger demographic. Our use of validated tools (KSS/WOMAC) and longitudinal assessments strengthens the robustness of our conclusions. The mechanistic basis for these associations may involve vitamin D's pleiotropic effects. Deficiency exacerbates sarcopenia and reduces type II muscle fiber recruitment, critical for post-TKA mobility [17]. Additionally, vitamin D modulates inflammatory cytokines (e.g., TNF-α, IL-6), which influence postoperative pain and tissue repair [18]. Our regression models confirmed that vitamin D status was an independent predictor, suggesting its role extends beyond confounding factors like obesity or age. Clinically, our findings support preoperative vitamin D screening and repletion. A threshold of ≥30 ng/mL was associated with optimal outcomes, consistent with Endocrine Society recommendations for musculoskeletal health [12]. Notably, 80% of our cohort had suboptimal levels, highlighting a modifiable risk factor. Future trials should evaluate whether supplementation (e.g., 2000-4000 IU/day) preoperatively improves outcomes, as seen in hip fracture studies [19]. Limitations include our single-center design and relatively short follow-up. Seasonal variations in vitamin D were accounted for but may not fully capture annual fluctuations. Additionally, we did not assess parathyroid hormone or bone turnover markers, which could provide mechanistic insights. Strengths include prospective data collection, blinded outcome assessments and rigorous adjustment for confounders.

## Conclusion:

A significant association between preoperative vitamin D deficiency and inferior functional outcomes in TKA patients is shown. Deficiency was prevalent (45%) and independently predicted poorer KSS and WOMAC scores at 6 months, even after adjusting for key confounders. Thus, we show the importance of routine vitamin D assessment and repletion in TKA candidates to optimize postoperative recovery. Future research should investigate the efficacy of targeted supplementation protocols.
